# Expansion of CD4 T Lymphocytes Expressing Interleukin 17 and Tumor Necrosis Factor in Patients with Major Depressive Disorder

**DOI:** 10.3390/jpm11030220

**Published:** 2021-03-19

**Authors:** Miguel Angel Alvarez-Mon, Ana Maria Gómez-Lahoz, Arancha Orozco, Guillermo Lahera, David Diaz, Miguel A. Ortega, Agustin Albillos, Javier Quintero, Enrique Aubá, Jorge Monserrat, Melchor Alvarez-Mon

**Affiliations:** 1 Department of Psychiatry and Medical Psychology, University Clinic of Navarra, Avda. Pío XII, 36, 31008 Pamplona, Spain; eauba@unav.es; 2 Department of Medicine and Medical Specialities, University of Alcala, 28871 Alcala de Henares, Spain; alahoz1199@gmail.com (A.M.G.-L.); guillermo.lahera@gmail.com (G.L.); david.diaz@uah.es (D.D.); miguel.angel.ortega92@gmail.com (M.A.O.); agustin.albillos@uah.es (A.A.); jorge.monserrat@uah.es (J.M.); mademons@gmail.com (M.A.-M.); 3 Department of Psychiatry and Mental Health, Hospital Universitario Infanta Leonor, 28031 Madrid, Spain; fjquinterog@salud.madrid.org; 4 Department of Psychiatry, University Hospital “Principe de Asturias”, 28805 Alcala de Henares, Spain; aranorsa@gmail.com; 5 CIBERSAM (Biomedical Research Networking Centre in Mental Health), 22807 Madrid, Spain; 6 Institute Ramon y Cajal for Health Research (IRYCIS), 28034 Madrid, Spain; 7 Department of Gastroenterology, University Hospital Ramon y Cajal, 28034 Madrid, Spain; 8 Biomedical Institute for Liver and Gut Diseases (CIBEREHD), Instituto de Salud Carlos III, Av. Monforte de Lemos, 3-5, 28029 Madrid, Spain; 9 Department of Legal and Psychiatry, Complutense University, 28040 Madrid, Spain; 10 Service of Internal Medicine and Rheumatology, Autoimmune Diseases University Hospital “Principe de Asturias”, 28805 Alcala de Henares, Spain

**Keywords:** major depressive disorder, CD4^+^ T lymphocytes, cytokines, interferon gamma, tumor necrosis factor, personalized medicine, precision medicine, translational research, clinical research

## Abstract

Background: We have investigated the distribution of the Th1, Th2 and Th17 subsets in circulating CD4^+^ T lymphocytes and their naïve (T_N_), effector (T_E_), central (T_CM_) and effector memory (T_EM_) activation/differentiation stages in patients with major depressive disorder (MDD). Methods: Thirty MDD patients and 30 healthy controls were studied. The counts of circulating CD4^+^ T lymphocytes and their distribution on the T_N_, T_E_, T_CM_ and T_EM_ activation/differentiation stages were analyzed by polychromatic flow cytometry. The intracytoplasmic interferon gamma (IFNγ), interleukin (IL)-4, IL-17A and tumor necrosis factor alpha (TNF-alpha) and membrane CD28 expression were also measured. The serum IFNγ, IL-4, Il-17A and TNF-alpha were measured by Luminex, respectively. Results: MDD patients had normal counts of CD4^+^ T lymphocytes and of their T_N_, T_CM_ and T_EM_ subsets but increased number and percentage of T_E_ CD4^+^ subset. CD4^+^ T lymphocytes had significantly enhanced percentage of cells that express IL-17 and TNF-alpha explained by the expansions found in the T_N_, T_CM_ and, T_EM_ and T_CM_, T_EM_ and T_E_ activation/differentiation stages, respectively. A selective increase in the percentages of T_CM_ and T_EM_ expressing IFNγ was also observed. We found a significant correlation between the percentages of CD4^+^ T lymphocytes expressing IFNγ and TNF-alpha in these patients. MDD patients showed increased serum levels of IL-17 and TNF-alpha, but normal IFNγ and IL-4 concentration. Limitations: the cross-sectional nature of the study could be considered a limitation. Conclusions: MDD patients have abnormal circulating CD4^+^ T lymphocytes with expansion of the IL-17 and TNF-alpha expressing cells as well as increased levels of circulating IL-17 and TNF-alpha.

## 1. Introduction

Major depressive disorder (MDD) is an exceedingly prevalent disease that causes significant disability worldwide [1]. Taking into account that approximately one-third of patients exhibit a poor response to MDD treatment, the need for more effective therapeutic strategies is a pressing medical objective [2]. Both experimental and human findings have underlined the relevance of abnormal immune-inflammatory response in the pathogenesis of depression [3]. Immunomodulation is a potential innovative therapy for MDD patients with promising results having been reported [4].

CD4^+^ T lymphocytes are critically important in the regulation of the immune response. CD4^+^ T lymphocytes are a phenotypical and regulatory diverse immune system cell population. This lymphocyte heterogeneity includes different patterns of cytokine secretion and stages of differentiation/activation [5,6,7]. CD4^+^ T lymphocyte subsets are characterized by their capacity to produce cytokines such as interferon (IFN)γ, interleukin (IL)-4 or IL-17A, and are subsequently referred to as Th1, Th2 and Th17, respectively [8]. Furthermore, based on their distinctive pattern of activation and effector functions, CD4^+^ T lymphocytes are categorized into different subsets according to their CCR7, CD27 or CD62L antigen surface expression. The critical difference in the expression of these membrane molecules is the kinesis of their loss or acquisition throughout the different T activation/differentiation stages [9]. Therefore, it has been proposed that CD4^+^ T lymphocyte subsets include CD45RA+CCR7+ naïve (T_N_), CD45RA-CCR7+ central memory (T_CM_), CD45RA-CCR7- effector memory (T_EM_) and CD45RA+CCR7- effector (T_E_) subsets [10]. T_N_ exhibit non-effector functions while T_CM_ show high rates of proliferation and express multiple effector molecules, such as cytokines, in response to antigen stimulation and less intensive activation requirements [11,12]. T_EM_ also expresses effector cytokines but has reduced proliferative capacity while T_E_ is at a final differentiation stage, exhibiting increased levels of cytokine production [13]. In addition to the different requirements for activation, proliferation and survival, T_N_, T_CM_, T_EM_ and T_E_ subsets also show a distinct capacity between them in terms of entering lymphoid and non-lymphoid inflamed tissues [14].

Although contradictory results have been published, there is a general consensus indicating that MDD patients have abnormal circulating levels of pro-inflammatory and regulatory cytokines [15]. Increased levels of tumor necrosis factor (TNF-alpha), a critical pro-inflammatory and regulatory cytokine, have been found in patients with MDD [16]. Furthermore, the serum levels IL-17A have also been found to be normal or elevated in MDD patients, with some reports also showing increased concentrations of serum IFNγ [17,18]. In this context, we have hypothesized that MDD patients could have an abnormal distribution of CD4^+^ T lymphocytes throughout the different stages of activation/differentiation, as well as abnormal patterns of cytokine production that might be involved in the pathogenesis of the immune dysfunction observed in MDD patients.

In this study, we have investigated the distribution of the Th1, Th2 and Th17 subsets in circulating CD4^+^ T lymphocytes and their T_N_, T_CM,_ T_EM_ and T_E_ activation/differentiation stages in patients with MDD. In order to avoid confounding factors, we selected a homogeneous population of 30 MDD patients for our study, one with no other concurrent diseases present that could be associated with immune system abnormalities. Simultaneously, we included 30 age, sex, body mass index (BMI), race and epidemiologically matched healthy controls (HCs). We also studied the serum levels of IFNγ, IL-4, IL-17 and TNF-alpha.

## 2. Materials and Methods

### 2.1. Inclusion and Exclusion Criteria

We recruited 30 patients diagnosed with MDD from the Departments of Psychiatry at both the Clinica Universidad de Navarra and the Hospital Universitario Príncipe de Asturias. The inclusion criteria were as follows: (a) a psychiatrist-confirmed diagnosis of MDD, single or recurrent, according to Diagnostic and Statistical Manual of Mental Disorders criteria, Fifth Edition (DSM-V) (American Psychiatric Association, 2013); (b) a minimum score of 14 points on the 17-item Hamilton Rating Scale for Depression (HRSD); and (c) an age between 18 and 65 years. Potential subjects were excluded for the following reasons: (1) an acute (exhibited in the last three months) or chronic bacterial or viral infection; (2) the use of steroids or any immunomodulatory pharmacotherapy in the past three months; (3) an autoimmune disease, a cardiovascular disease (e.g., hypertension and ischemic heart disease), or a hematopoietic, lung, hepatic, or renal disorder; (4) an endocrine or metabolic disease (e.g., diabetes mellitus and hypercholesterolemia) or a body-mass index (BMI) higher than 30; (5) a history of malignancy; (6) immunodeficiency or malnutrition; (7) pregnancy or lactation; (8) concomitant psychiatric disorder, evaluated using the MINI International Neuropsychiatric Interview [19]. Simultaneously, we studied 30 sex-, age-, and BMI-matched HCs belonging to the same epidemiological area.

The ethics committees of the Clinica Universidad de Navarra and the Hospital Universitario Príncipe de Asturias both gave their approval for this study. Prior to their enrollment, all participating individuals gave their written consent only after having the nature and characteristics of the study fully explained to them.

### 2.2. Isolation of Peripheral Blood Mononuclear Cells

Peripheral blood mononuclear cells (PBMC) were obtained from heparinized venous blood and were separated by Ficoll-Hypaque (Lymphoprep^TM^, Axis-Shield, Oslo, Norway) gradient centrifugation. They were then resuspended in RPMI-1640 with 10% heat-inactivated fetal calf serum (Gibco, Thermofisher, Madrid, Spain), 25mM HEPES and 1% penicillin-streptomycin (Biowhittaker, Verviers, Belgium). Cell enumeration was performed as previously described. The PBMCs of each patients or control were adjusted to 1 10^6^ cells/mL prior to antibody staining.

### 2.3. Surface CD28 Lymphocyte Staining

CD28 expression in the activation/differentiation stages CD4^+^ T lymphocytes were studied in PBMCs by flow-cytometry; 5 × 10^5^ PBMCs were incubated with the next surface-labeled monoclonal-antibodies, CD4-PercP, CD28-PECY7 (Becton-Dickinson, San Jose CA, USA), CD8-Alexa405, CD45RA-APC (Caltag, San Francisco, CA, USA), CCR7-APCAlexa780 (eBioscience, San Diego, CA, USA) and CD3 (Alexa-700). Samples were washed twice and acquired in a FacsAria-II flow-cytometer and were analyzed using FacsDiva 5.0 and Flow-Jo 10.0 software. Results of CD28 expression were analyzed with respect the total of CD4 T lymphocytes (CD3+CD4+) and their activation stages T_N_ (CD3+CD4+CD45RA+CCR7+), T_MC_ (CD3+CD4+CD45RA-CCR7+), T_EM_ (CD3+CD4+CD45RA-CCR7-) and T_E_ (CD3+CD4+CD45RA+CCR7-) activation/differentiation stages.

### 2.4. In Vitro Culture

The spontaneous and stimulated T-lymphocyte subset expression of IFNγ, IL-4, IL-17A, and TNF-alpha was assessed by in vitro intracytoplasmic staining assay. Then, 1 × 10^6^ PBMCs were cultivated and stimulated with 50 ng/mL phorbol-12-myristate-13-acetate (PMA, Sigma-Aldrich, Merck, Barcelona, Spain) plus 1 μg/mL ionomycin (Calbiochem, LaJolla, CA, USA) in the presence of 2 mM monensin (Merck, Barcelona, Spain) for 6 h. Spontaneous cytokine expression was determined in parallel cultures in the absence of exogenous stimuli.

### 2.5. Intracellular Lymphocyte Cytokines Assay

T-lymphocytes were analyzed in PBMCs by nine-color flow-cytometry. PBMCs were incubated with the next surface-labeled monoclonal-antibodies, CD3-PercP, CCR7-PECY7 (Becton-Dickinson, San José, CA, USA), CD8-Alexa405, CD45RA-APC (Caltag, San Francisco, CA, USA) and CD27-APCAlexa780 (eBioscience, San Diego, CA, USA).

For intracytoplasmic staining, PBMCs were fixed and permeabilized (Fix and Perm, Caltag, San Francisco CA, USA), and cytokines were stained with IL-4-PE, IFNγ Alexa700 and IL-17A-FITC (Becton-Dickinson, San José, CA, USA). All samples were stained with a dead cell-discriminator simultaneously with antibody addition (fixable aqua dead cell stain kit for 405 nm excitation; Molecular Probes, Eugene, OR). Samples were acquired in a FacsAria-II flow-cytometer and were analyzed using FacsDiva 5.0 and Flow-Jo 10.0 software.

### 2.6. Cytokines Serum Levels

Serum samples from MDD patients and HCs were aliquoted, identified and labeled, and frozen at −80 °C. Then, they were thawed and cytokines were quantified using the high sensitivity human MILLIPLEX^®^ kit (Merck, Barcelona, Spain) to simultaneously measure IFNγ, IL-4, TNF and IL-17A (Millipore, Merck, Barcelona, Spain) following the manufacturer’s instructions and revealing the results by Luminex (MAGPIX^®^ system). The measured cytokines had the following sensitivity limits (0.48 pg/mL for IFNγ, 1.12 pg/mL for IL-4, 0.33 pg/mL for IL-17A and 0.16 for TNF-alpha). The results were analyzed using Analyst 5.1 software MILLIPLEX^®^.

### 2.7. Statistical Analysis

Analyses were performed using SPSS-22 software (SPSS-IBM, Armonk, NY, USA). Since most variables did not fulfill the normality hypothesis, the Mann–Whitney U-test for non-parametric data was used to analyze differences between groups, and Pearson correlation coefficient was used for the association between indicated continuous variables. The significance level was set at *p* < 0.05.

## 3. Results

### 3.1. Patient Demographic Characteristics

The demographic data and clinical characteristics of the 30 MDD patients and 30 HCs included in the study are shown in Table 1. Concerning the demographic characteristics compared between MDD patients and HCs, significant differences were only found for the variable of employment status. The patient group included 19 females and 11 males, ranging from 27 to 53 years of age. The duration of their depressive episode before enrollment in the study was 16.12 ± 2.85 weeks; 17 of these patients (56.7%) had suffered at least one previous MDD episode. The mean value of the HRSD was 15.95 ± 1.25 at the time patients were recruited. Moreover, 10% of the patients presented psychotic (delusional) symptoms during the current episode of study.

All patients received pharmacological treatment according to their doctors’ discretion: 30 (100%) received antidepressant medications, 28 (93.3%) anxiolytics or hypnotics, 5 (16.7%) mood stabilizers, and 10 (33.3%) atypical antipsychotics. In addition, 28 patients (93.3%) received combination pharmacotherapy consisting of at least 2 different types of medication in 19 patients (63.3%) and at least 3 different types of medication in the other 9 patients (30%). None of the patients were treated with electroconvulsive therapy (ECT).

### 3.2. MDD Patients Show Increased Counts of the T_E_ CD4 T Lymphocyte Subset

We studied the circulating counts of CD4^+^ T lymphocytes and their T_N_, T_CM_, T_EM_ and T_E_ activation/differentiation stages in 30 MDD patients and 30 sex-, age-, body mass index-, ethnicity- and smoking status-matched HCs. There were no significant differences in the number of circulating CD4^+^ T lymphocytes between MDD patients and HCs (Table 2). Furthermore, we investigated the distribution of the subsets in CD4^+^ T cells from MDD patients and HCs. There were no significant differences in the counts and percentages of the T_N_, T_CM_ and T_EM_ CD4^+^ subsets between patients and HCs. However, MDD patients had a significant increase in the number and percentage of T_E_ CD4^+^ lymphocytes.

In addition, we did not find any significant differences in the number and percentage of cells expressing or lacking CD28 in the total of CD4^+^ T lymphocytes, nor in their T_N_, T_CM_ and T_EM_ subsets among MDD patients and HCs (Table 3). Nonetheless, we found a significant increase in the number and percentage of the minority T_E_ subsets in MDD patients with respect to HCs.

### 3.3. MDD Patients Show an Expansion of Circulating Th17 and TNF^+^ CD4^+^ T Lymphocytes

**Figure 1:**
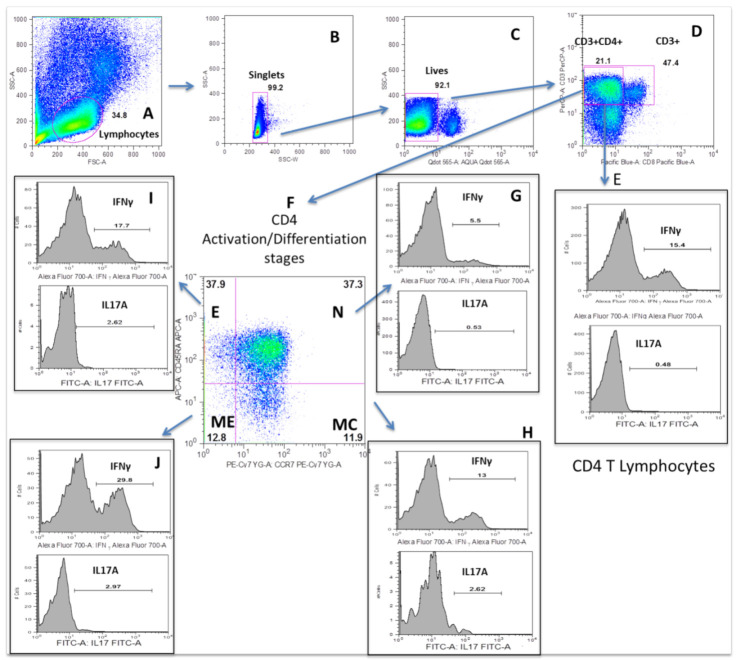
Dot plots represent the flow cytometry gating strategy and histograms of the intracellular interleukin (IL)-17A, interferon (IFN)γ, tumor necrosis factor alpha (TNF-alpha) and IL-4 expression by total circulating CD4^+^ T lymphocytes and T_N_, T_CM_, T_EM_ and T_E_ subsets in a representative case of MDD. The first row of dot plots represents the selected gates and percentages to obtain the total CD4^+^ T lymphocytes and T_N_, T_CM_, T_EM_ and T_E_ subsets in the presence of PMA (50 ng/mL) stimulation for 4 h. Histograms represent the percentages of IL-17A, IFNγ, TNF-alpha and IL-4 producing cells in the indicated CD4^+^ T lymphocytes subsets. Gating strategy: (**A**) Selection of lymphocytes by size (FSC) and complexity (SSC). (**B**) Exclusion of doublets. (**C**) Exclusion of dead cells. (**D**) Negative selection of CD4^+^ T lymphocytes using CD3^+^CD8^-^ cells. (**E**) Expression of IFNγ and IL-17A in total CD4^+^ T lymphocytes. (**F**) Analysis of the activation/differentiation states of CD4^+^ T lymphocytes. (**G**–**J**) Expression of IFNγ and IL-17A in T_N_, T_CM_, T_EM_ and T_E_ lymphocytes.

**Figure 2:**
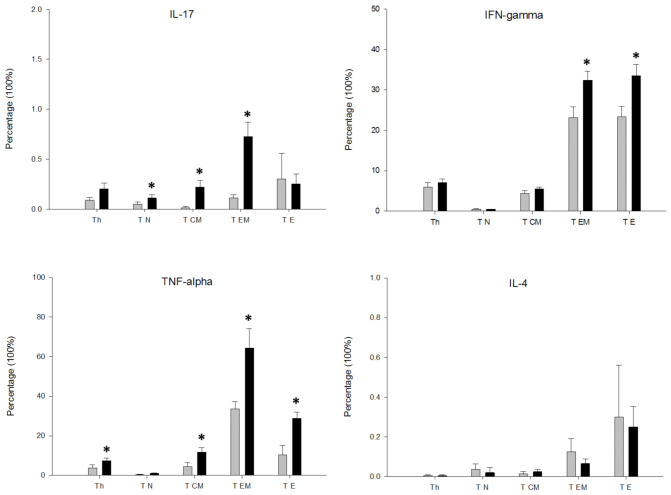
Percentage of IL-17A, IFNγ, TNF-alpha and IL-4 expression by total circulating CD4^+^ T lymphocytes and T_N_, T_CM_, T_EM_ and T_E_ subsets in MDD patients and healthy controls after stimulation with PMA. Percentage of cells (y axis) that express the indicated cytokine by total CD4^+^ T lymphocytes and their T_N_, T_CM_, T_EM_ and T_E_ subsets (x axis) in MDD patients (black rectangles plots) and HCs (gray rectangles plots). * Significant difference between MDD and HCs for the indicated variable.

We investigated the intracellular expression of IFNγ, IL-4, IL-17A and TNF-alpha in the total CD4^+^ T lymphocyte population and in the T_N_, T_CM_, T_EM_ and T_E_ differentiation/activation stages among MDD patients and HCs after PMA stimulation. Figure 1 shows the flow cytometry gating strategy and the histograms of the intracellular IFNγ, IL-4, IL-17 and TNF-alpha expressions shown by the total circulating CD4^+^ T lymphocytes and the T_N_, T_CM_, T_EM_ and T_E_ differentiation/activation stages of a representative case of MDD. We found that the percentage of the total CD4^+^ T lymphocyte population that expressed IL-17 and TNF-alpha in MDD patients was significantly higher than in the HCs (Figure 2). The increased percentage of CD4^+^ T lymphocytes expressing IL-17 and TNF-alpha in MDD patients was explained by the significantly enhanced percentages found in both the T_N_, T_CM_, T_EM_ and T_CM_, T_EM_, T_E_ activation/differentiation stages, respectively. We also found a selective increase in the percentages of T_E_ and T_EM_ expressing IFNγ in MDD patients.

**Figure 3:**
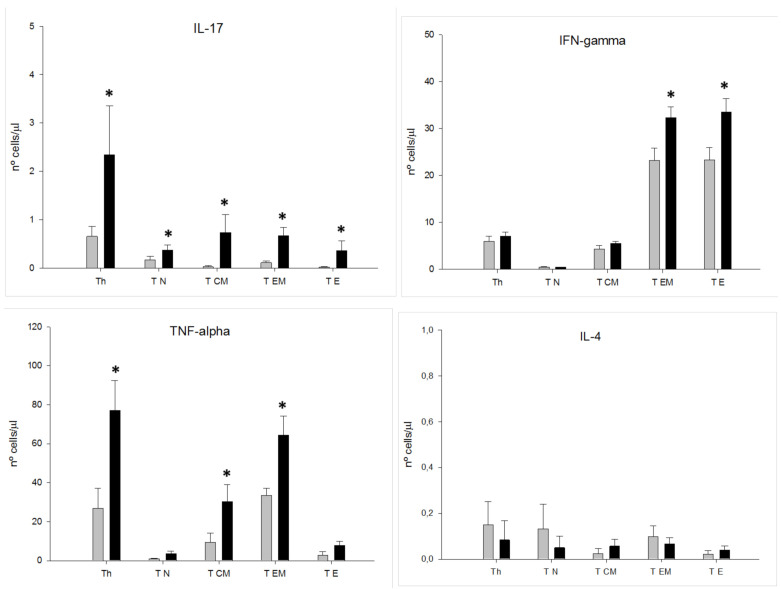
Circulating CD4^+^ T lymphocytes and T_N_, T_CM_, T_EM_ and T_E_ subsets that are able to express IL-17A, IFNγ, TNF-alpha, and IL-4 in MDD patients and HCs. Absolute number (cells/μL) (y axis that are able to express the indicated cytokine by total CD4^+^ T lymphocytes and their T_N_, T_CM_, T_EM_ and T_E_ subsets (x axis)) in MDD patients (black rectangles plots) and HCs (gray rectangles plots). * Significant difference between MDD and HCs for the indicated variable.

In both groups of subjects, we calculated the potential number of circulating CD4^+^ T lymphocytes that could express IFNγ, IL-4, IL-17 and TNF-alpha by multiplying the total number of T lymphocytes and the four different activation/differentiation stages of CD4^+^ T lymphocyte subsets by the percentage of cells expressing the analyzed cytokines after PMA stimulation in the defined subsets (Figure 3). We observed that the number of circulating CD4^+^ T lymphocytes and their T_N_, T_CM_, T_EM_ and T_E_ differentiation/activation stages that could express IL-17 was significantly increased in MDD patients compared to those levels found in the HCs. Among MDD patients, we also found that the number of circulating CD4^+^ T lymphocytes that could express TNF-alpha was significantly enhanced, which is explained by a selective increase in the number of T_CM_ and T_EM_ differentiation/activation stages that could produce this cytokine. We also observed that although the number of circulating CD4^+^ T lymphocytes that could express IFNγ was normal, those in the T_EM_ and T_E_ differentiation/activation stages were significantly increased.

**Figure 4:**
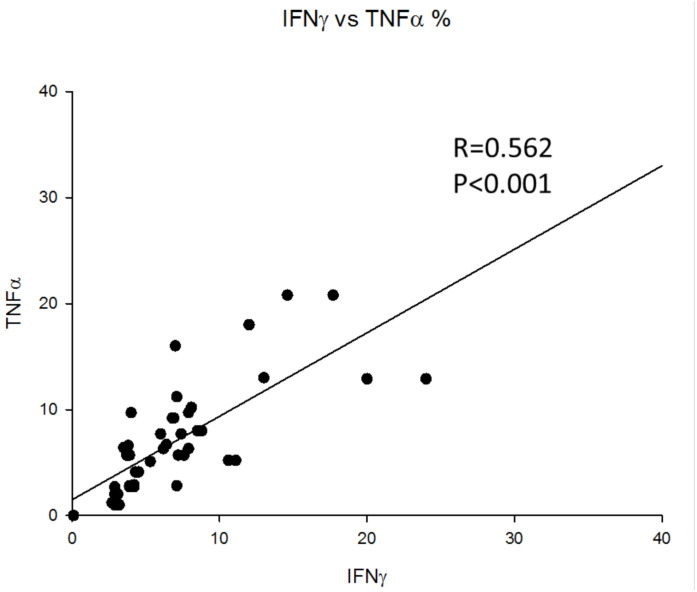
Correlations between the percentages of IFNγ and TNF-alpha expression by CD4^+^ T lymphocytes in MDD patients. Pearson correlation coefficient between percentages of expression of IFNγ and TNF-alpha was 0.562 (*p* < 0.0001).

Additionally, we investigated the potential correlations between the expression of IFNγ, IL-17 and TNF-alpha shown by CD4^+^ T lymphocytes among MDD patients (Figure 4). More specifically, we observed a significant correlation between the percentages of cells expressing IFNγ and TNF-alpha in CD4^+^ T lymphocytes in these patients. However, we did not find a statistical correlation between the percentages of cells expressing either IL-17 and TNF-alpha (*p* = 0.073) or IL-17 and IFNγ (*p* = 0.32) in CD4^+^ T lymphocytes. Furthermore, the percentage of CD4^+^ T lymphocytes that had a double cytokine expression was less than 0.01%.

**Figure 5:**
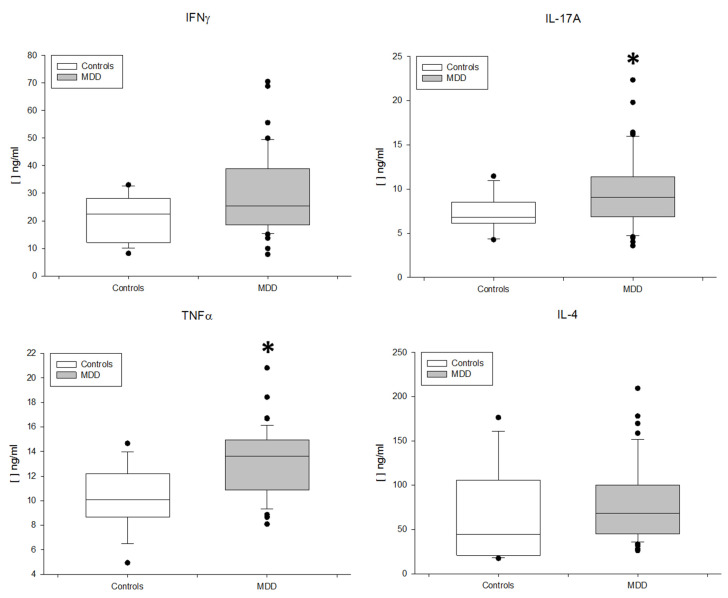
Circulating serum levels of IL-17A, IFNγ, TNF-alpha and IL-4 in MDD patients (black rectangles plots) and HCs (gray rectangles plots). Serum concentrations (pg/μL) (y axis) of IFNγ, IL-17A, IL-4 and TNF-alpha. * Significant difference between MDD and HCs for the indicated variable.

Finally, we also measured the circulating levels of IFNγ, IL-4, IL-17 and TNF-alpha in MDD patients and HCs (Figure 5). MDD patients had significantly increased levels of circulating IL-17 and TNF-alpha, but normal IFNγ and IL-4 serum concentrations.

## 4. Discussion

In this paper, we have shown that MDD patients have abnormally functioning CD4^+^ T lymphocytes with an expansion of the Th-17 and TNF-alpha subsets. This functional bias of the CD4^+^ T-cell population is explained by a significantly increased percentage of CD4^+^ T lymphocytes expressing IL-17 and TNF-alpha at the T_N_, T_CM_, T_EM_ and T_CM_, T_EM_, T_E_ stages of activation/differentiation, respectively. MDD patients show increased levels of circulating IL-17 and TNF-alpha, but normal IFNγ and IL-4 serum concentrations.

An abnormal immune response within a systemic inflammatory environment has been involved in the pathogenesis of depression [20]. However, the mechanisms involved in the immune dysregulation described in MDD patients remain elusive. CD4^+^ T lymphocytes play a critical role in the regulation of the natural and adaptative immune responses. The activity of the different Th1, Th2 and Th17 CD4^+^ T lymphocytes is crucial for the induction of a proinflammatory and effector pattern immune response [7]. Our data clearly shows an increased inclination towards Th17 differentiation in the CD4^+^ T lymphocyte circulating population of MDD patients. The increased frequency of IL-17 expression is observed in the T_N_, T_CM_ and T_EM_ differentiation/activation stages of CD4^+^ T lymphocytes. We have also found a selective IFNγ overexpression in the T_CM_ and T_EM_ CD4^+^ T lymphocyte differentiation/activation stages. Conflicting results regarding the Th1, Th2 and Th17 counts and CD4^+^ T lymphocyte differentiation/activation stages have been previously described in MDD patients [18,21,22,23,24]. Several reasons that are not mutually exclusive may explain these variabilities. The differences may be due to different cellular preparations, experimental models and measurement technologies employed for the identification of CD4^+^ T lymphocyte phenotypes and functions, as well as the clinical characteristics of the MDD patients and controls who were analyzed. In this study, we have employed a precise cytometric strategy for the analysis of CD4^+^ T lymphocytes. We have focused our research on a homogenous population of patients with MDD experiencing persistent symptomatology for an interval lasting between 10 and 20 weeks. In designing our study to discover the relevance of the impact of MDD, we included subjects with persistent MDD symptoms in spite of their pharmacological treatment, therefore excluding those with a rapid response to treatment. Low-grade inflammation has been shown to influence neurotransmission, that is an important determinant in MDD pathogenesis and response to treatment [25]. In fact, previous reports show that patients with MDD with an activated inflammatory state show reduced responses rates to antidepressants [26]. Furthermore, with this study strategy, we avoided the described modulation of Th2 CD4^+^ T lymphocytes associated with effective antidepressant treatment, no matter the antidepressant prescribed [27]. Additionally, in order to prevent any potential interference with CD4^+^ T lymphocyte function from concomitant or previous diseases and/or treatments, we applied precise exclusion criteria favoring the homogeneity of the patient population and the absence of potential causes of interference. We studied as controls a matched sex, age, race and BMI group of HCs from a similar epidemiological area. To our knowledge, this is the first evidence of an increased frequency in CD4^+^ T lymphocytes expressing TNF-alpha in MDD patients, as explained by the expansion observed in the lymphocyte T_CM_, T_EM_ and T_E_ differentiation/activation stages.

As previously discussed, in addition to their pattern of cytokine production, CD4^+^ T lymphocytes are a heterogeneous population with different stages of differentiation/activation, patterns of circulation and tissue infiltration [10,14]. Notably, the number of CD4^+^ T_N_ able to express IL-17A^+^ is increased in MDD patients. This finding suggests an abnormal bias of non-antigen activated CD4^+^ T lymphocytes from these patients towards IL-17A production, which is also observed in antigen-promoted T_CM_ and T_EM_ CD4^+^ T lymphocytes. These results are aligned with the recently described expansion of memory and Th17 CD4^+^ T lymphocytes in patients with depression exhibiting a high risk of suicide [28]. The relevance of the predisposition and acquired activation of CD4^+^ T lymphocytes to express cytokines is supported by the observation of opposing results with respect to IFNγ and TNF-alpha production in MDD patients [29]. The increasing counts of CD4^+^ T lymphocytes producing TNF-alpha and IFNγ in MDD patients were mainly focused on the T_CM_, T_EM_, T_E_ and T_EM_ and T_E_ CD4^+^ T lymphocytes, respectively. Different mechanisms might be involved in these functional findings, the activating microenvironment and/or the genetic characteristics of the patients. There is evidence supporting the relevance of the cytokine microenvironment in the differentiation of naïve T lymphocytes into Th1, Th2 and Th17 subsets [30]. Consequently, it is possible to suggest that MDD may be associated with an intrinsic IL-17A^+^T_N_ differentiation. However, antigens and cytokines favor Th1 differentiation and TNF-alpha expression with a predominance of T_M,_ T_EM_ and T_E_ CD4^+^ T lymphocyte activation. Furthermore, the plasticity of Th17 to differentiate into Th1 has been shown.

Our results assist in explaining the claimed relevance of IL-17 and TNF-alpha in the pathogenesis of MDD [31,32]. According to our findings, the expansion of both Th17 and TNF-alpha expressing CD4^+^ T lymphocytes is observed in MDD patients, with this expansion not homogenously distributed. We did not find significant correlation between the percentages of CD4^+^ T lymphocytes expressing IL-17 and TNF-alpha. In contrast, we observed a significant correlation between the percentages of CD4^+^ T lymphocytes expressing IFNγ and TNF-alpha. Thus, it is possible to suggest that MDD patients show different stages or types of CD4^+^ T lymphocyte disturbance as reflected in the preferential pattern of IL-17 or TNF-alpha expression. These results may explain the previously described heterogeneity evidenced among studies on CD4^+^ T lymphocyte cytokine production in MDD patients [33].

Although the etiology of mood disorders is heterogeneous, the pathogenic relevance of immunological alterations in MDD is supported by the therapeutic interventions with anti-inflammatories and immunomodulators. Anti-inflammatory agents may be effective for the treatment of depression, at least for a significant proportion of patients presenting baseline inflammatory activation [34]. Furthermore, there is evidence supporting biologics specifically targeting individual cytokines (mainly TNF-alpha and IL-6) as effective in reducing depressive symptoms in a subset of MDD patients [4,35]. The clinical response to anti-TNF drugs appears to be related to the pattern of inflammatory-immune disturbances in these patients [36]. Our findings support that in a subset of MDD patients, an expansion of Th-17 CD4^+^ T lymphocytes correlated with increased IL-17 levels, independently of TNF-alpha expression. Thus, it is possible to suggest that IL-17 might be considered a potentially new target for the personalized treatment of depression [37,38]. Moreover, an analysis of the frequency of Th17 and TNF-alpha expressing CD4^+^ T lymphocytes might serve as a complementary biomarker for the selection of biological treatments.

The potential dynamic variability of the CD4^+^ T lymphocyte alterations in MDD patients found in this study has not been established because our design did not include any patient follow-up. However, our results do not support a maintained and chronic activation of CD4^+^ T lymphocytes in cases of depression. It is known that chronic long-term inflammatory diseases are associated with a reduction in the number of T_N_ stages and an increase in the percentage of CD28- CD4^+^ T lymphocytes [39]. In our study, we have observed a normal number and percentage of T_N_ and CD28 expression in CD4^+^ T lymphocytes in MDD patients. However, we have found a significant increase in the number and percentage of CD28- T_E_ subset in MDD patients that is considered as a senescence marker [40]. Thus, a potentially temporal variation in CD4^+^ T lymphocyte disturbances might be associated with depressive clinical activity.

However, our work does have limitations as it was designed to be a translational, cross-sectional study of MDD patients. Nevertheless, we have included a homogenous population with persistent MDD symptomatology in spite of pharmacological treatment and without any disease potentially interfering with the immune system. It is important to note, that antidepressants might have an immunomodulatory effect [41,42]. Thus, future studies designed to establish the pattern of association between the disturbance of Th CD4^+^ T lymphocytes and the clinical evolution of depression should include patients without pharmacological treatment. The number of patients included in our study might be considered as being reduced. However, the aim of this study was not to establish the potential association of the alterations of the activation/maturation and Th pattern of cytokine expression by CD4^+^ T lymphocytes and the clinical phenotype of MDD.

Further studies have to be conducted for the translational analysis of the potential association of the specific CD4^+^ T lymphocyte abnormalities observed in this study with the different clinical manifestations of the disease. Additionally, a selective number of biological parameters have to be studied within a wide population of patients throughout the clinical evolution of the disease. Finally, although requiring further confirmation from future transnational research, the rational and promising findings behind our study provide evidence of CD4^+^ T lymphocytes potentially functioning as biomarkers for therapeutic targets in MDD patients.

## Figures and Tables

**Table 1:** Baseline characteristics of participants.

	MDD	HC	*p* Value
Socio-demographic			
Age, mean (SD)	43.26 (13.14)	41.45 (11.46)	0.35
Sex (% female)	19 (63.3%)	21 (70%)	0.98
Currently employed and active n (%)	13 (59.1%)	27 (90%)	<0.01
College degree n (%)	16 (53.3%)	20 (66.6%)	0.45
Past History			
Family history of depression n (%)	17 (56.7%)	12 (40%)	0.38
Family history of other psychiatric disorder n (%)	22 (77.3%)	14 (46.6%)	0.22
Health characteristics and somatic comorbidities			
BMI, mean (SD)	26.74 (5.41)	25.5 (5.36)	0.51
Smoking n (%)			0.13
- Never	12 (40%)	8 (26.6%)	
- Occasionally	8 (26.7%)	15 (50%)	
- Everyday	10 (33.3%)	7 (23.3%)	
Drinking n (%)			0.3
- Never	7 (23.3%)	6 (20%)	
- Occasionally	20 (66.7%)	22 (73.3%)	
- Everyday	3 (10%)	2 (6.6%)	

**Table 2:** Circulating CD4^+^ T lymphocytes in MDD patients. Circulating counts and percentages of CD4^+^ T lymphocytes and of their naïve (T_N_), central (T_CM_), effector memory (T_EM_) and effector (T_E_) subsets in MDD patients and HCs. * represent *p* < 0.05 between MDD patients and HCs.

	HC (%)	MDD (%)	HC (Cells/L)	MDD (Cells/L)
Th	75.22 ± 5.77	76.45 ± 2.44	1038.99 ± 222.94	1037.98 ± 88.55
T N	60.06 ± 6.94	62.31 ± 2.88	693.05 ± 191.81	621.58 ± 51.74
T CM	32.37 ± 6.12	29.29 ± 2.31	292.05 ± 77.33	326.43 ± 51.12
T EM	6.64 ± 1.27	7.01 ± 0.79	48.70 ± 8.95	76.15 ± 11.75
T E	0.91 ± 0.39	1.41 ± 0.34*	5.13 ± 1.67	14.06 ± 2.72 *

**Table 3:** Circulating counts and percentages of CD28-CD4^+^ T lymphocytes and of their T_N_, T_CM_, T_EM_ and T_E_ subsets in MDD patients and HCs. * represent *p* < 0.05 between MDD patients and HCs.

	HC (%)	MDD (%)	HC (Cells/L)	MDD (Cells/L)
Th CD28-	13.66 ± 4.78	13.52 ± 2.63	149.38 ± 62.90	129.57 ± 30.02
T N CD28-	10.63 ± 3.28	9.96 ± 1.81	232.05 ± 79.57	244.89 ± 38.32
T CM CD28-	14.43 ± 6.55	5.04 ± 1.05	25.05 ± 9.99	16.97 ± 5.00
T EM CD28-	24.87 ± 10.63	16.61 ± 3.10	7.85 ± 3.14	14.12 ± 3.49
T E CD28-	37.27 ± 11.45	48.91 ± 6.70*	2.74 ± 1.49	8.78 ± 2.56 *

## Data Availability

The datasets generated for this study are available on request to the corresponding author.
